# Data on farmers' determinants of manure and inorganic fertiliser use in the semi-arid Ethiopian Rift Valley

**DOI:** 10.1016/j.dib.2017.08.042

**Published:** 2017-09-01

**Authors:** Shiro Mukai

**Keywords:** Fertiliser, Manure, Integrated soil fertility management, Subsampling method, Semi-arid Ethiopian lowlands

## Abstract

This article contains the data on farmers' determinants of binary choices for manure use (i.e., manure is used or unused) and fertiliser use (i.e., fertiliser is used or unused) at their fields in semi-arid northern Ethiopian Rift Valley. The data includes (i) a schematic diagram that represents local farmers' distinctions of the crop field types in terms of the distance from their houses and soil fertility and (ii) a table that describes a representative farmer's crop sequences and soil fertilisation methods in two consecutive years. Details about the literature review of the previous case studies on farmers' determinants of manure application technique adoption conducted in some parts of sub-Saharan Africa where cattle dung is used for manure are also summarized in a table. A table shows descriptive statistics of the independent variables used in the empirical analyses. Summary statistics of 4 binomial logit models and 4 multinomial logit models are indicated in a table, which represent model fit. Last two tables exhibited in this article show the logit analyses.

**Specifications Table**

**Table:** 

Subject area	*Agricultural Economics*
More specific subject area	*Innovative technology adoption study*
Type of data	*Figure, tables, text file*
How data was acquired	*Interviews with farmers, field observation, semi-structured questionnaire survey, binomial logit analysis*, *multinomial logit analysis*
Data format	*Raw and analysed*
Experimental factors	*524 plot data were collected from farmers’ fields through a semi-structured questionnaire survey*
Experimental features	*Logit analyses to examine farmers’ determinants of soil fertilisation options*
Data source location	*Two districts (Adama and Boset) in the semi-arid northern Ethiopian Rift Valley*
Data accessibility	*Data is with this article*

**Value of the data**

• The data include the figure that describes local farmers' distinctions of the crop field types in terms of the distance from their houses and soil fertility in semi-arid northern Ethiopian Rift Valley.
• The data include the table that shows a representative farmer's crop sequences and soil fertilisation methods.
• These data are a benchmark for farmers' determinants of manure application and fertiliser use at their crop fields in Ethiopian lowlands including semi-arid Ethiopian Rift Valley.
• These data can be compared to the similar type of analyses conducted in other areas.

## 1 Data

**Fig. 1:**
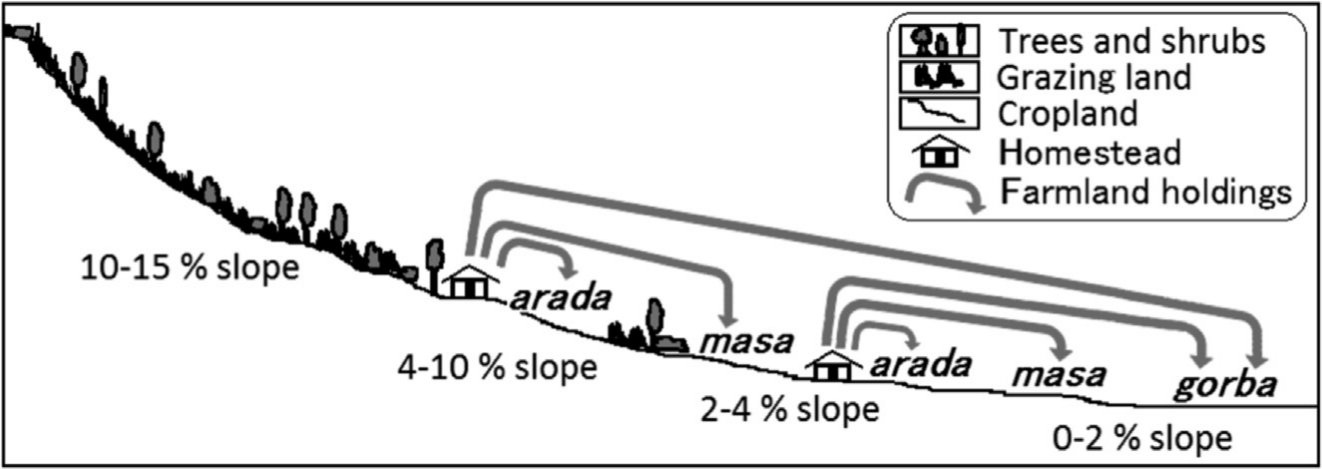
Positional relationships between homesteads, *aradas*, *masas*, and *gorbas* (drawing by Shiro Mukai).

This article includes a schematic diagram representing local farmers' distinctions of the crop fields in terms of the distance from their houses and soil fertility (Fig. 1), table that summarizes the literature review of the previous case studies on determinants of manure application techniques conducted in other parts of sub-Saharan Africa where cattle dung is used for manure (Table 1), table describing a representative farmer's crop sequences and soil fertilisation methods in 2011 and 2012 (Table 2). Table 3 shows descriptive statistics of the independent variables used in the empirical analyses. The logit analysis data include the summary statistics of four binomial logit models and four multinomial logit models, which represent model fit (Table 4), variable coefficients of the two binomial logit models, which are selected as appropriate models in Table 4 (Table 5), and average marginal effects of the three multinomial logit models, which are selected as appropriate models in Table 4 (Table 6).

**Table 1:** Case studies on determinants of manure application techniques conducted in some parts of SSA.

No. and sources	Fertilisation^a^	Crops			Dependent		Model^g^	Dependent variables used^h^							
		Target^b^	Food crops^c^	Cash crops^d^	Data^e^	Type^f^		*zones*	*gender*	*training*	*off-farm*	*farm*	*livestock*	*labour*	*market*
1 [6]	M	AC	Mi, So	Pe, Cow	HH	Bi	B. L.	^**^(+)	*ns*(+)	^***^(+)	–	–	–	*ns*(+)	–
2 [7]	M	AC	Ma	Ma, Be	HH	Bi	B. L.	–	–	^**^(+)	–	^**^(–)	^**^(+)	–	–
3 [8]	M	AC	Ma, So	Cow, Gr	HH	Bi	B. L.	^***^(+)	–	–	–	–	*ns*(+)	^*^(+)	–
4 [9]	M	AC	Ba, Wh	Wh, Be	Plot	Bi	M. L.	–	^**^(–)	^**^(+)	–	*ns*(+)	^**^(+)	*ns*(–)	*ns*(+)
5 [10]	M	AC	Ca, Ma	Ho, Ve	HH	Bi	B. L.	–	–	^**^(+)	–	^**^(+)	–	–	–
6 [11]	M	Ma	Ma, Be	Sug, Cot	HH	Bi	M. L.	–	–	*ns*(±)	*ns*(–)	*ns*(–)	–	^*^(+)	–
7 [12]	M	AC	Ma, Wh	Ma, Tef	HH	Bi	B. L.	–	*ns*(–)	–	–	–	^***^(+)	–	–
8 [13]	M	AC	Wh, Ma	Wh, Ba	HH	Bi+In	B. P.+T.	–	*ns*(+)	^***^(+)	*ns*(+)	*ns*(–)	^***^(+)	^*^(+)	*ns*(-)
9 [14]	M + F	Ma	Ma, Be	Tea, Cof	HH	Bi	B. L.	–	–	^***^(–)	^*^(–)	*ns*(+)	*ns*(+)	*ns*(+)	–
10 [15]	M + F	Ma	Ma, Wh	Ma, Tef	HH	Bi	B. L.	^**^(+)	–	–	–	–	^**^(+)	*ns*(–)	–
11 [16]	M + F	Ma	Ma, Be	Tea, Cof	HH	Bi	M. L.	–	^**^(–)	^*^(+)	*ns*(+)	^**^(+)	^***^(+)	^***^(–)	–
12 [17]	M + F	AC	Mi, So	Gr, Cot	HH	Bi	B. P.	–	–	–	–	–	^**^(+)	–	–
13 [18]	M + F	AC	–i	–	Plot	Bi	M. L.	–	*ns*(+)	*ns*(+)	*ns*(-)	–	*ns*(–)	^***^(+)	–
14 [19]	M + F	AC	Ma, Be	Tea, Cof	Plot	Bi	MV. P.	–	^**^(+)	–	^***^(+)	^**^(+)	^*^(+)	^***^(+)	–
15 [20]	M + F	AC	Ma, Be	Tea, Cof	HH	In	Tob.	–	*ns*(+)	–	^**^(–)	*ns*(+)	–	^*^(+)	*ns*(–)
16 [21]	M + F	AC	Ma, So	Cow, Gr	HH	Bi	M. L.	–	–	^**^(+)	*ns*(+)	*ns*(+)	*ns*(+)	*ns*(+)	–
17 [22]	M + F	AC	Ma, So	Chat, Cof	Plot	Bi	TS. P.	–	*ns*(–)	*ns*(+)	–	^***^(–)	*ns*(+)	^*^(+)	–
18 [23]	M + F	AC	Ma, Wh	Ma, Tef	HH	Bi	M. L.	^**^(+)	–	*ns*(+)	*ns*(+)	*ns*(+)	^**^(+)	^**^(+)	–

a M, manure; M + F, manure and fertiliser.

b Crops targeted in each study. AC, all crops; Ma, maize.

c Main food crops grown in each study area. Cow, cowpea; Cof, coffee; Cot, cotton; Gr, groundnuts; Py, pyrethrum; Sug, sugarcane; Ve, vegetables.

d Main cash crops grown in each study area. Ba, barley; Be, beans; Ca, cassava; Chat, *Catha edulis*; Mi, millet; Po, potato; So, sorghum; Wh, wheat.

e Type of the data collected as dependent variables. HH, household data; Plot, plot data.

f Type of dependent variables. Bi, binary choices of adoption or not adoption; In, intensity of use.

g Econometric model used. B. L., binomial logit; B. P., binomial probit; M. L., multinomial logit; 2SLS, two-stage least squares; MV. P., multivariate probit; Tob., Tobit; TS. P., two-stage probit.

h –, not used; *ns*, not significant; *P<0.1; **P<0.05; ***P<0.01; Signs (+,–) in parentheses are the signs of the variable coefficients.

i 851 household and plot data were collected from 123 areas across Uganda [18]. Thus main food and cash crops in each of the study areas were not described here.

**Table 2:** Crop sequences and soil fertilisation practices of Mr. TY over two years, 2011 and 2012.

Field no.	Type of the field	Area of the plot (ha)	Distance from farmers' house (m)	Cropping (fertilisation option and application level)	
				2011	2012
1	*Arada*^a^	0.06	0	Maize (household waste input)	Maize (household waste input)
2	*Arada*	0.5	10	Maize (household waste input)	Maize (household waste input)
3	*Masa*	0.5	50	Tef (compost 0.125 Mg)^b^	Sorghum (no fertilisation)
4	*Masa*	0.25	100	Haricot bean (compost 0.2 Mg)^c^	Tef (compost 0.2 Mg + fertiliser)
5	*Masa*	0.5	200	Tef (compost 0.1 Mg + fertiliser)	Wheat (fertiliser)
6	*Masa*	0.25	200	Tef (compost 0.1 Mg + fertiliser)	Barley (compost 0.1 Mg)
7	*Masa*	1.0	400	Wheat (compost 0.1 Mg + fertiliser)	Tef (compost 0.3 Mg + fertiliser)
8	*Masa*	0.25	700	Barley (fertiliser)	Tef (fertiliser)
9	*Masa*	0.25	1000	Tef (fertiliser)	Haricot bean (no fertilisation)

a A backyard field.

b Mr. TY did not input fertiliser in 2011 but used it when he cultivated tef in 2010.

c Mr. TY applied approximately 0.16 Mg compost in 2006.

**Table 3:** Descriptive statistics of the independent variables used in the empirical analyses.

Variables	Expected sign	Fertilisation options (*n*=524)^a^			
		*no fertilisation* (*n*=43)	*manure*	*fertiliser*	*manure+fertiliser*
			(*n*=220)	(*n*=156)	(*n*=105)
Socioeconomic characteristics of the sample farm households					
*zone* (1=MM, 0=MD sub-areas)	+	0.21^a^	0.55^b^	0.53^b^	0.46^b^
*gender* (1=male, 0=female)	±	0.86^ns^	0.88^ns^	0.85^ns^	0.93^ns^
*training* (1=received, 0=otherwise)	+	0.67^ns^	0.56^ns^	0.53^ns^	0.66^ns^
*off-farm* (1=engaged, 0=otherwise)	±	0.35^ns^	0.30^ns^	0.28^ns^	0.26^ns^
*fertiliser* (1=used, 0=otherwise)	±	0.00^a^	0.00^a^	1.00^b^	1.00^b^
*farm* (Total farmland holding; ha)	±	1.76^ab^	2.08^ab^	1.82^a^	2.48^b^
*livestock* (Livestock ownership level; TLU)^b^	+	2.47^a^	3.42^b^	2.68^a^	4.32^c^
*labour* (Family and permanent labour force; persons)^c^	±	3.45^ab^	3.57^ab^	3.42^ab^	3.80^b^
*market* (Distance from the nearest market centre; km)	±	0.97^a^	2.47^b^	1.74^c^	2.41^b^
					
Biophysical characteristics of the sample farm plots					
*crop* (1=CMCFs, 0=OCMFs)		0.91^a^	1.00^b^	0.02^c^	0.00^c^
*plotsize* (Size of the sample plot; ha)	+	0.51^a^	0.22^b^	0.39^c^	0.39^c^
*distance* (Distance from the HH house to the plot; m)	±	1453^a^	81^b^	1402^a^	790^c^
0 m < ≤ 100 m^d^		5 (12%)	191 (87%)	30 (19%)	24 (23%)
100 m < ≤ 1000 m		22 (51%)	28 (13%)	67 (43%)	52 (50%)
1000 m <		16 (37%)	3 (1%)	56 (36%)	29 (28%)
Type of the sample plot *arada*		12 (28%)	220 (100%)	0 (0%)	0 (0%)
*masa*		0 ( 0%)	0 (0%)	153 (98%)	105 (100%)
*gorba*		31 (72%)	0 (0%)	3 (2%)	0 (0%)

a Different superscript letters indicate statistically significant differences between the fertilisation options (*P*<0.05). *ns* not significant.

b TLU, Tropical livestock unit; *livestock* (TLU)=cattle ownership level (TLU)×(1−fuel use rate (%)/100)+other livestock ownership level (TLU). The fuel use rate indicates what percentage of dung produced by the cattle owned by the sample household was consumed for fuel.

c Converted to adult (from 16 to 65 years old) labour force equivalent.

d Segments of *distance* (m).

**Table 4:** Summary statistics of the four binomial logit models (A1, A2, A3, and A4) and four multinomial logit models (B1, B2, B3, and B4).

Model	LL	BIC	AIC	*χ*^2^	Df	*p*-value	*R*^2^	%	SSR
A1	−30.99	123.10	83.97	139.42	10	<0.0001	0.69	95	9.1
A2	−148.01	357.10	318.02	52.66	11	<0.0001	0.15	71	50.6
A3	−213.43	508.26	452.86	263.85	12	<0.0001	0.38	81	70.1
A4	−217.43	509.99	458.85	255.86	11	<0.0001	0.37	81	71.5
B1	−44.93	212.36	133.86	162.41	20	<0.0001	0.66	94	255.1
B2	−165.31	453.13	374.62	59.45	20	<0.0001	0.15	69	1972.9
B3	−219.11	663.64	510.23	874.27	33	<0.0001	0.67	81	2232.4
B4	−455.98	1118.60	977.97	400.53	30	<0.0001	0.31	64	2504.2

LL, log-likelihood; *χ*^2^, Wald *χ*^2^; Df, the degrees of freedom; *R*^2^, pseudo *R*^2^; %, % correctly predicted; SSR, the sum of squared residuals.

**Table 5:** Variable coefficients of the two binomial logit models (models A1 and A2).

	A1 (CMCF subdataset)	A2 (OCMF subdataset)	A3 (pooled dataset with *crop*)	A4 (pooled dataset without *crop*)
Dependent variable *manure*: 1=used, 0=not used				
Independent variables				
*zone*	−0.13 (0.88)	−0.58*(0.30)	−0.19 (0.26)	−0.17 (0.26)
*gender*	−0.76 (1.02)	0.65 (0.50)	0.51 (0.37)	0.50 (0.36)
*training*	−0.41 (0.74)	0.38 (0.29)	0.11 (0.25)	0.12 (0.25)
*off‐farm*	1.11 (0.84)	−0.16 (0.33)	−0.09 (0.28)	−0.09 (0.28)
*fertiliser*	omitted	omitted	1.06 (1.13)	−1.83***(0.27)
*crop*	not used	not used	3.00***(1.14)	not used
*farm*	0.29 (0.40)	0.21**(0.09)	0.23***(0.09)	0.23***(0.09)
*livestock*	0.71***(0.23)	0.21***(0.06)	0.23***(0.06)	0.21***(0.06)
*labour*	0.00 (0.30)	−0.10 (0.09)	−0.06 (0.08)	−0.05 (0.08)
*market*	0.28 (0.27)	0.14 (0.08)	0.19***(0.07)	0.19*** (0.07)
*plotsize*	−5.18***(1.69)	0.37 (0.80)	−2.22***(0.58)	−2.16***(0.58)
*Distance*	−0.00***(0.00)	-0.00***(0.00)	−0.00***(0.00)	−0.00***(0.00)

Numbers in parentheses are standard error. In both models A1 and A2, variable *fertiliser* was omitted due to collinearity with the dependent variable. The analyses of models A3 and A4 were for reference.

* *P*<0.1.

** *P<*0.05.

*** *P<*0.01.

**Table 6:** Average marginal effects of the three multinomial logit models (models B1, B2, and B3).

Dependent variables	B1 (CMCF subdataset)			B2 (OCMF subdataset)			B3 (pooled dataset)			
	*no fertil- isation*	*manure*	*fertiliser*	*no fertil- isation*	*fertiliser*	*manure+fertiliser*	*no fertil- isation*	*manure*	*fertiliser*	*manure+fertiliser*
Independent variables										
*zone*	−0.044	0.044	0.000	−0.008	0.112*	−0.104*	−0.030	0.022	0.059	−0.051
	(0.754)	(0.033)	(1.000)	(0.018)	(0.058)	(0.058)	(0.020)	(0.020)	(0.029)	(0.029)
*gender*	0.040	−0.039	−0.000	−0.016	−0.106	0.122	0.004	−0.020	−0.045	0.062
	(0.309)	(0.041)	(0.999)	(0.021)	(0.097)	(0.097)	(0.025)	(0.025)	(0.050)	(0.049)
*training*	0.024	−0.027	0.000	−0.009	−0.068	0.076	0.006	−0.014	−0.030	0.038
	(0.873)	(0.030)	(0.999)	(0.016)	(0.057)	(0.056)	(0.018)	(0.018)	(0.029)	(0.028)
*off‐farm*	−0.018	0.020	−0.000	−0.003	0.034	−0.030	−0.002	0.010	0.010	−0.015
	(0.568)	(0.030)	(0.998)	(0.018)	(0.066)	(0.065)	(0.018)	(0.017)	(0.033)	(0.033)
*crop*	not used	not used	not used	not used	not used	not used	−0.369	0.519	1.419	−1.569
							(38.36)	(38.11)	(280.25)	(289.48)
*farm*	−0.012	0.012	0.000	−0.003	−0.038**	0.042**	−0.007	0.006	−0.020**	0.021**
	(0.146)	(0.014)	(1.000)	(0.007)	(0.018)	(0.018)	(0.008)	(0.007)	(0.009)	(0.009)
*livestock*	−0.024	0.024***	0.000	0.005	−0.045***	0.039***	−0.009**	0.012	−0.023***	0.019***
	(0.027)	(0.008)	(0.998)	(0.004)	(0.012)	(0.011)	(0.004)	(0.007)	(0.006)	(0.006)
*labour*	−0.001	0.001	−0.000	−0.009	0.023	−0.013	−0.001	0.001	0.007	−0.007
	(0.198)	(0.011)	(0.998)	(0.009)	(0.019)	(0.018)	(0.006)	(0.006)	(0.009)	(0.009)
*market*	−0.022	0.022*	0.000	−0.005	−0.025	0.029**	−0.014*	0.011	−0.012	0.015**
	(0.056)	(0.012)	(0.998)	(0.007)	(0.015)	(0.015)	(0.008)	(0.008)	(0.008)	(0.007)
*plotsize*	0.130	−0.131**	0.000	0.001	−0.070	0.070	0.058**	−0.066	−0.029	0.037
	(0.391)	(0.053)	(0.998)	(0.058)	(0.160)	(0.157)	(0.031)	(0.045)	(0.077)	(0.079)
*distance*	0.000	−0.000***	0.000	0.000	0.000***	−0.000***	0.000***	−0.000*	0.000***	−0.000***
	(0.000)	(0.000)	(0.998)	(0.000)	(0.000)	(0.000)	(0.000)	(0.000)	(0.000)	(0.000)

Numbers in parentheses are standard error.

* *P*<0.1.

** *P<*0.05.

*** *P<*0.01.

## 2 Experimental design, materials and methods

### 2.1 Study area

Adama and Boset districts in Oromia region, Ethiopia, are classified into five agroecological sub-zones (tef zone, maize zone, semi-pastoral zone, sorghum and tef zone, and wheat and tef zone) [1]. Subsistence crop (sorghum, maize, and barley) and cash crop (tef, wheat, haricot bean, and vegetables) fields are mixed in all zones. The two districts are categorised into mid-altitude dry (MD) sub-area and mid-altitude moist (MM) sub-area in terms of major maize growing areas in Ethiopia [2].

### 2.2 Sample

The following two-step procedures were used to select sample plots: in the first step, we set a goal to select 150 households from each maize growing sub-area. The target numbers of households were equally split by the number of sub-zones in each sub-area: four and two sub-zones in the MD and MM sub-areas, respectively, and were allocated to each sub-zone. Semi-structured questionnaires were prepared for interviewing randomly selected household heads in November and December 2012. After eliminating questionnaires with invalid data, we had data from 146 and 173 household heads living in the MD and MM sub-areas, respectively. It was found these 146 and 173 household heads had 313 continuous maize cropping fields (CMCFs; 151 for MD and 162 for MM sub-areas) and 302 other than maize cropping fields (OCMFs; 131 for MD and 171 for MM sub-areas). In the second step, we randomly selected 262 CMCFs (131 for MD and 131 for MM sub-areas) and 262 OCMFs (131 for MD and 131 for MM sub-areas) from these 313 CMCFs and 302 OCMFs to match the numbers of the plot data between CMCFs and OCMFs and between MD and MM sub-areas. The total number of the plot data became 524 (262 CMCFs+262 OCMFs).

### 2.3 Empirical models

An preliminary field survey conducted in 2011 showed that the CMCFs (*n*=262) had three fertilisation options: (i) no fertilisation (*n*=39), (ii) manure application (*n*=220), and (iii) fertiliser use (*n*=3), while the OCMFs (*n*=262) had three fertilisation options: (i) no fertilisation (*n*=4), (ii) fertiliser use (*n*=153), and (iii) both compost and fertiliser use (*n*=105). The following two empirical exercises were conducted by using different econometric models:

(i) To analyse farmers' determining factors in binary manure use options (dependent variable, *manure*: 1=used, 0=not used), two binomial logit models were formulated for CMCF and OCMF subdatasets (model A1 and model A2, respectively). Another two binomial logit models were created with and without variable *crop* (main cropping system to which the sample plot belonged; 1=CMCFs, 0=OCMFs) for the pooled dataset (model A3 and model A4, respectively); and
(ii) To assess the farmers' determinants of four fertilisation options (dependent variable, *fertilisation*: 1=no fertilisation, 2=manure application, 3=fertiliser use, 4=both manure and fertiliser use), two multinomial logit models were built for the CMCF and OCMF subdatasets (model B1 and model B2, respectively). Another two multinomial logit models were formulated with variable *crop* (model B3) and without variable *crop* (model B4) for the pooled dataset. Hausman test or Small-Hsiao test [3] was conducted to verify the independence of irrelevant alternatives (IIA) hypothesis.

Independent variables selected in this study were based upon the literature on technology adoption studies of manure/fertiliser use (Table 1, Table 3). To select appropriate models for further analyses, indicators of the optimum model selection for logit models [4] including the log-likelihood, McFadden's pseudo-*R*^2^, Akaike's information criterion (AIC), Bayesian information criterion (BIC), and the % correctly estimated values were examined. To test the validity of the subsampling method, the sum of squared residuals obtained from the pooled dataset and subdatasets to test the equality of coefficients were compared between the models [5]. Stata 13.0 (StataCorp LP) was used for the empirical calculations.

### 2.4 Logit analyses

Summary statistics (Table 4) showed the indicators of the optimum model selection for logit models and the sum of squared residuals (SSR) obtained from the four binomial logit models (A1, A2, A3, and A4) and four multinomial logit models (B1, B2, B3, and B4). The binomial logit and multinomial logit analyses were shown in Table 5, Table 6, respectively.
